# Oxidative genomic or genotoxic stress in neurodegeneration: Mechanisms and therapeutic avenues

**DOI:** 10.3934/Neuroscience.2025020

**Published:** 2025-09-12

**Authors:** Taslim Uddin

**Affiliations:** Department of Biotechnology & Genetic Engineering, Jahangirnagar University, Savar, Dhaka-1342, Bangladesh

**Keywords:** oxidative stress, DNA damage, neurodegeneration, mitochondria, therapeutic targets, repair mechanisms, ROS, neuroinflammation

## Abstract

Neurons are especially vulnerable because of their high metabolic activity and limited ability to repair damaged DNA. Oxidative genotoxic stress (OGS), which arises from the buildup of short-lived, highly reactive molecules called reactive oxygen species (ROS), can damage neuronal DNA and compromise antioxidant defense mechanisms in neurons. OGS induces considerable forms of DNA damage, including genomic instability, DNA strand breaks (single or double), DNA base modifications such as 8-oxoguanine, and epigenetic changes, leading to compromised neuronal functions. Moreover, OGS is a silent player in mitochondrial DNA damage and mitochondrial dysfunction. Therefore, ROS-mediated OGS is pivotal for initiating and advancing several neurodegenerative diseases, such as Alzheimer's disease (AD), Parkinsonism (PD), and Huntington's disease (HD). However, there is a significant gap in deciphering the molecular pathways involved in OGS-mediated development of neurodegenerative diseases. Hence, this study focused on the molecular mechanisms by which OGS causes neurodegeneration, with a focus on the contributions of neuroinflammation, mitochondrial dysfunction, and defective DNA repair pathways. Additionally, new therapeutic approaches, such as mitochondrial-targeted medications, antioxidant therapies, gene editing tools such as CRISPR/Cas9, and biomarkers for the early diagnosis of these oxidative diseases, have been assessed. A thorough comprehension of these processes opens exciting possibilities for focused treatments in neurodegenerative illnesses.

## 1. Introduction

Neurodegenerative disorders are characterized by the progressive deterioration of neuronal cells and the eventual loss of neurons, leading to a gradual decline in the cognitive and motor functions of the central nervous system (CNS). These conditions predominantly include Alzheimer's disease (AD), Parkinson's disease (PD), Huntington's disease (HD), and amyotrophic lateral sclerosis (ALS), which represent the most prevalent forms within this category. The most commonly known form of AD is the predominant kind of illness globally, constituting 60%–80% of all dementia cases and impacting approximately 24 million individuals worldwide. A community-based study in the United States indicated that the prevalence may reach 50% among individuals over 85 years of age [1]. AD is the root cause of dementia in older adults because of the accumulation of microtubule-associated amyloid-beta (Aβ) and tau protein neurofibrillary tangles in the brain, leading to progressive impairments in memory, thinking, and behavior. Posttranslational modifications of acetylation and hyperphosphorylation are the etiological factors of Aβ and tau protein production in AD patients.

PD, a universally prevalent neurodegenerative disorder, has been implicated with OGS/ROS as a central pathomechanism. PD has more than doubled in 26 years, from approximately 2.5 million patients in 1990 to almost 6.1 million (5.0–7.3) in 2016. It is believed that population aging is partially responsible for this trend [2]. The neuropathology of PD is principally distinguished by the progressive degradation of dopaminergic neurons in the region of the brain called the substantia nigra pars compacta (SNpc) and the creation of Lewy bodies that are still alive. Aggregated forms of α-synuclein (α-syn) constitute the primary constituent of Lewy bodies, which are widely acknowledged as the fundamental mechanism responsible for this neurodegenerative process. In addition, multiple sclerosis (MS), another form of chronic and inflammatory neurodegenerative disease of the CNS, affects more than 2.8 million individuals globally [3].

OGS has emerged as a potential etiological agent for the development of neurodegenerative diseases. OGS arises from the overabundance of short-lived, highly reactive molecules, called reactive oxygen species (ROS), and defective antioxidant defense mechanisms in cells, thereby causing DNA damage in cells and leading to genetic mutations and other genetic abnormalities, including genomic instability, defective DNA repair pathways, and mitochondrial dysfunctions. Under normal physiological conditions, ROS production and bioclearance are strongly controlled by a balanced antioxidant equilibrium, which is maintained with a variety of antioxidant defenses, such as the use of enzymatic scavengers, such as catalase (CAT), superoxide dismutase (SOD), and glutathione peroxidase. Hence, the effects of ROS-mediated OGS on neurons play a vital role in disease development. In addition, genetic and environmental factors linked to age-related neurodegenerative and neuropsychiatric illnesses have been extensively studied. The accumulation of chronic DNA damage, the activation of DNA damage response (DDR) signaling, pathological neuronal cell death, and senescence (biological aging) are associated with genomic instability, a term for genetic mutations or alterations in nucleic acid sequences. Therefore, these neurodegenerative illnesses and mental disorders develop over time as these defensive responses, or DDR pathways, become dysregulated due to aging or environmental factors [4].

Neurotoxins, including microbial toxins, chemical pollutants, heavy metals, cigarette smoke, and harmful gases, are other contributing factors to OGS-mediated neurodegenerative diseases. For example, gliotoxin (GTX) and ochratoxin A (OTA), which are natural fungal toxins, cause significant ROS production and downregulation of antiapoptotic genes, including *Bcl2*, leading to micronucleus formation, chronic neuronal inflammation, and impaired DNA repair with an aberrant cell cycle in neurons [5]. Micro and nanoplastics, which are toxic environmental pollutants with poor biodegradability, are another form of neurotoxin and are reported to cause significant oxidative stress, resulting in the dysfunction of biological and cellular processes, including apoptosis, endoplasmic reticulum stress, DNA damage, and inflammation in neurons [6].

In addition, lead toxicity, caused by the heavy metal lead (Pb), which is known to be a neurotoxin, also plays a vital role in ROS-mediated oxidative stress. Alterations in MDA and GSH levels, CAT activity, and altered expression of the *hsp70* and *ache* genes indicate significant DNA damage in the brain [7]. Furthermore, genotoxic substances (such as chemicals and radiation) can harm DNA both chemically and structurally. In some situations, they have a severe impact on genome integrity by triggering the oxidation of DNA bases, which interferes with fundamental bioprocesses such as transcription, transduction, and replication, eventually leading to cell death [8]. For example, endogenous genotoxins, including reactive nitrogen species (RNS), aldehydes, and alkylators, cause persistent and relentless genetic damage through OGS [9]. Endogenous DNA damage arises from hydrolysis, oxidation, alkylation, and base mismatches, whereas exogenous DNA damage is caused by ionizing radiation (IR), UV, and different chemical substances [10].

The mechanisms by which OGS influences neuronal susceptibility remain unclear, despite its significance in neurodegeneration. This review outlines the molecular pathways connecting oxidative stress-mediated OGS development with DNA damage and neurodegeneration. This study critically evaluates innovative therapies for enhancing DNA repair, enhancing mitochondrial protection, and exerting anti-inflammatory effects aimed at addressing oxidative DNA damage. This review integrates current knowledge to elucidate essential molecular insights and potential treatments for neurodegenerative diseases.

## 2. Molecular cross-talk of OGS-induced DNA damage: A silent killer for neurons

ROS and RNS are inescapable physiological byproducts that function as double-edged swords inside the biological framework, including nucleic acids, proteins, and lipids [11]. These compounds can enhance the function of signaling molecules under controlled conditions; however, when present in excess, they can damage the organic structure owing to their oxidizing properties. Neuronal tissue is resistant to ROS and RNS, with a unique response to DNA damage, regulation of the immune system, and control of inflammatory pathways. In neurodegenerative diseases, the whole system is disrupted, particularly by OGS-induced DNA damage, including DNA base changes, strand breakage, and abasic sites in neurons. Major DNA base modifications produced by oxidative stress include 8-oxoguanine (8-oxoG) and DNA strand breaks (single- or double-stranded). 8-oxoG is the most frequent biomarker for 8-oxo-7,8-dihydroguanine, the main guanine oxidation product found in genomic DNA. When ROS are created inside cells and react with DNA, 8-oxoG is produced at a rate of at least several hundred lesions per human cell per day, even under typical physiological circumstances [12]. This rate increases even more when there is reactive stress [13], resulting in the erroneous pairing of 8-oxoG with adenine (A), leading to an elevated frequency of replication errors. In addition, after a base is excised from DNA, a gap is created, which is called an abasic site or an AP (apurinic/apyrimidic) site. The inability of DNA repair mechanisms to effectively address severe damage loads results in multiple adverse outcomes.

The misincorporation of dATP by DNA polymerases, influenced by lesion templates, results in DNA alterations, especially in individuals with a mutated MUTYH gene, which is responsible for removing adenine bases from 8-oxoG/A mispairs [14]. The second detrimental consequence of chromosomal biomarkers is the erroneous bypass of the lesion by RNA polymerase II complexes during transcription, leading to RNA mutagenesis and the subsequent synthesis of abnormal proteins [15]. Ultimately, 8-oxoG reduces the transcriptional output of the affected gene even when a solitary lesion is adequate to elicit a substantial impact (8-oxoG reduces the transcriptional output by stalling RNA polymerase II during elongation and promoting mispairing with adenine, which leads to transcriptional mutagenesis). In addition, the recruitment of base excision repair proteins to the lesion competes with the transcription machinery, further lowering gene expression [16]. Single-strand breaks (SSBs) are the predominant type of DNA lesion resulting from base hydrolysis and oxidative degradation [17],[18]. Although they occur infrequently, stochastic mistakes during DNA replication can result in single-nucleotide changes, and ROS can induce oxidative DNA lesions such as 8-oxoG [19]. Exogenously caused lesions can be both mutagenic and cytotoxic. For example, UV radiation causes helix-distorting lesions such as cyclobutane pyrimidine dimers [20]. The continuation of neuronal loss in a wide range of human neurodegenerative illnesses is attributed to DNA damage, specifically DNA double-strand breaks (DSBs), according to recent research. This is not surprising, as the high metabolic activity and nonproliferative nature of neurons make them vulnerable to DNA damage. Nevertheless, it is unclear whether DSBs are the primary cause of neuronal damage in a disease or if they occur only as the illness worsens [21]. In most cases, DSBs are destructive and lethal types of genomic damage, causing neuronal cell death if they are left unrepaired or fixed incorrectly. In the case of cellular growth and division, these unrepaired DSBs can be a potential danger for cellular damage [22],[23], as unrepaired DSBs can cause mutations, deletions, and chromosomal translocations [24].

In contrast to proliferating cells, which can employ sister chromatids for error-free DSB repair through a crucial mechanism called homologous recombination (HR), postmitotic neurons rely on error-prone DSB repair mechanisms via nonhomologous end joining (NHEJ) [25]. Consequently, DSBs may significantly impair neuronal function and viability. Recent data indicate that the effects of DSBs and repair extend beyond cellular stress and pathological situations, as previously believed, and instead affect basic neuronal physiological processes. The intriguing findings of Madabhushi and colleagues support the theory that neural activity causes DSBs to occur on the promoters of a subset of early-response genes, which are essential for learning, memory, and modifications to synapses [26].

Exposure to residual oil fly ash (ROFA) led to notable disruptions in mitochondrial respiration, including diminished coupling efficiency, reduced respiratory capacity, and elevated proton leakage. These alterations coincided with a decrease in the mitochondrial membrane potential. Both NADPH oxidase (NOX), a membrane-associated enzyme complex, and mitochondria have been identified as key contributors to superoxide anion (O₂•−) generation. These data suggest that ROFA exposure directly activates macrophages, triggering an inflammatory response and enhancing reactive oxygen species (ROS) production through NOX and mitochondrial pathways. This oxidative stress undermines the antioxidant defense system and potentially contributes to mitochondrial dysfunction [27].

### 2.1. OGS-induced mitochondrial damage in neurons

**Figure 1.**
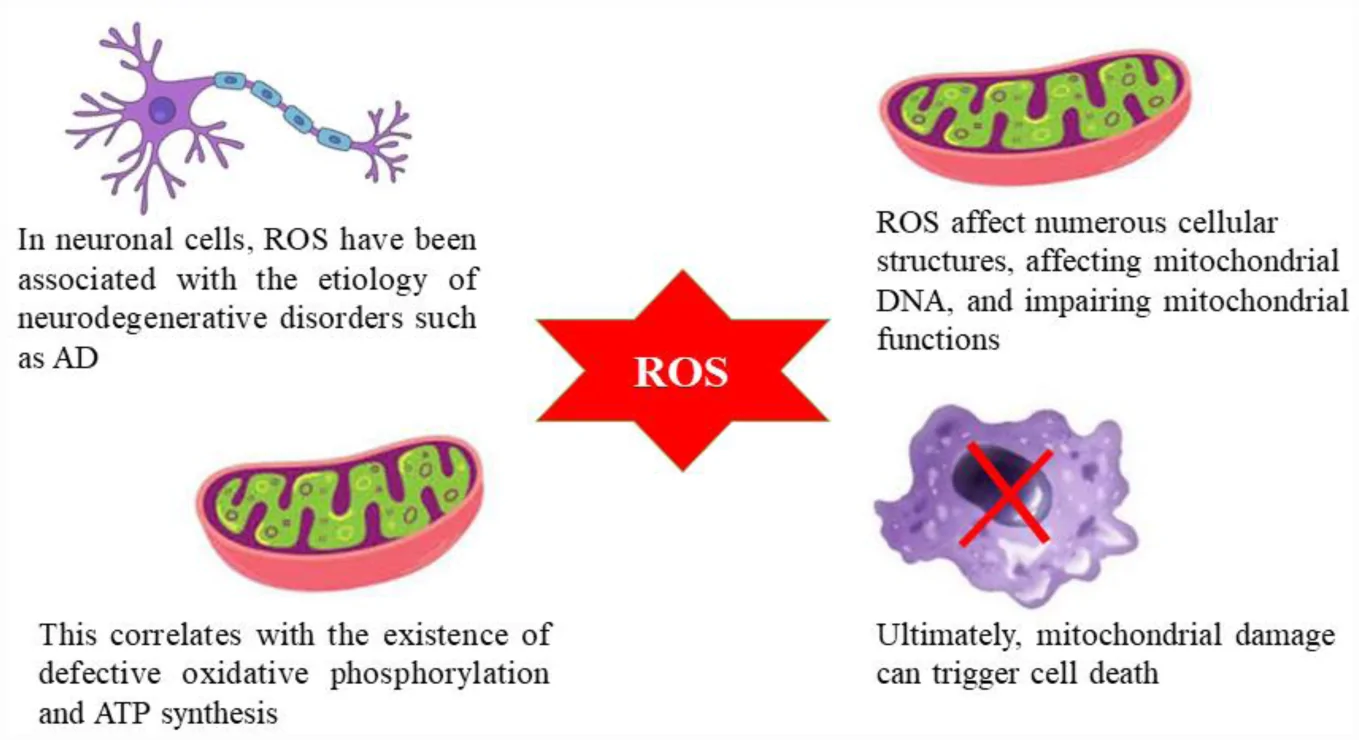
OGS-induced mitochondrial damage in neurons.

OGS-induced mitochondrial impairment in neuronal cells has been associated with the etiology of multiple neurodegenerative disorders, including Alzheimer's disease, Parkinson's disease, and amyotrophic lateral sclerosis, since dysfunctional mitochondria further intensify oxidative stress [28]. Mitochondrial DNA (mtDNA) is particularly susceptible to oxidative stress, and continued oxidative damage to mtDNA results in mutations within the mitochondrial genome. These mutations can subsequently hinder the functionality of respiratory chain complexes, resulting in a reduction in ATP synthesis and an exacerbation of ROS production [29]. This constitutes the essence of the vicious cycle of mitochondrial impairment. Energy shortfalls are especially detrimental to neurons, which require substantial energy to sustain membrane potentials, facilitate neurotransmission, and perform other essential processes [30],[31]. Mitochondria are essential for regulating intracellular calcium concentrations. Oxidative stress and mitochondrial impairment can disrupt this function, resulting in elevated cytoplasmic Ca^2+^ concentrations. This may initiate a series of events, including the activation of deleterious enzymes and the subsequent onset of apoptosis (programmed cell death) [32],[33]. Oxidative stress can trigger the opening of the mPTP, a nonselective channel in the inner mitochondrial membrane. This results in the dissipation of the mitochondrial membrane potential, mitochondrial enlargement, and the release of proapoptotic proteins, ultimately leading to cell death [34]. These pathological events are illustrated in Figure 1, which depicts OGS-induced mitochondrial damage in neurons.

### 2.2. OGS-mediated damage to DNA repair pathways

#### 2.2.1. Base excision repair (BER)

**Figure 2.**
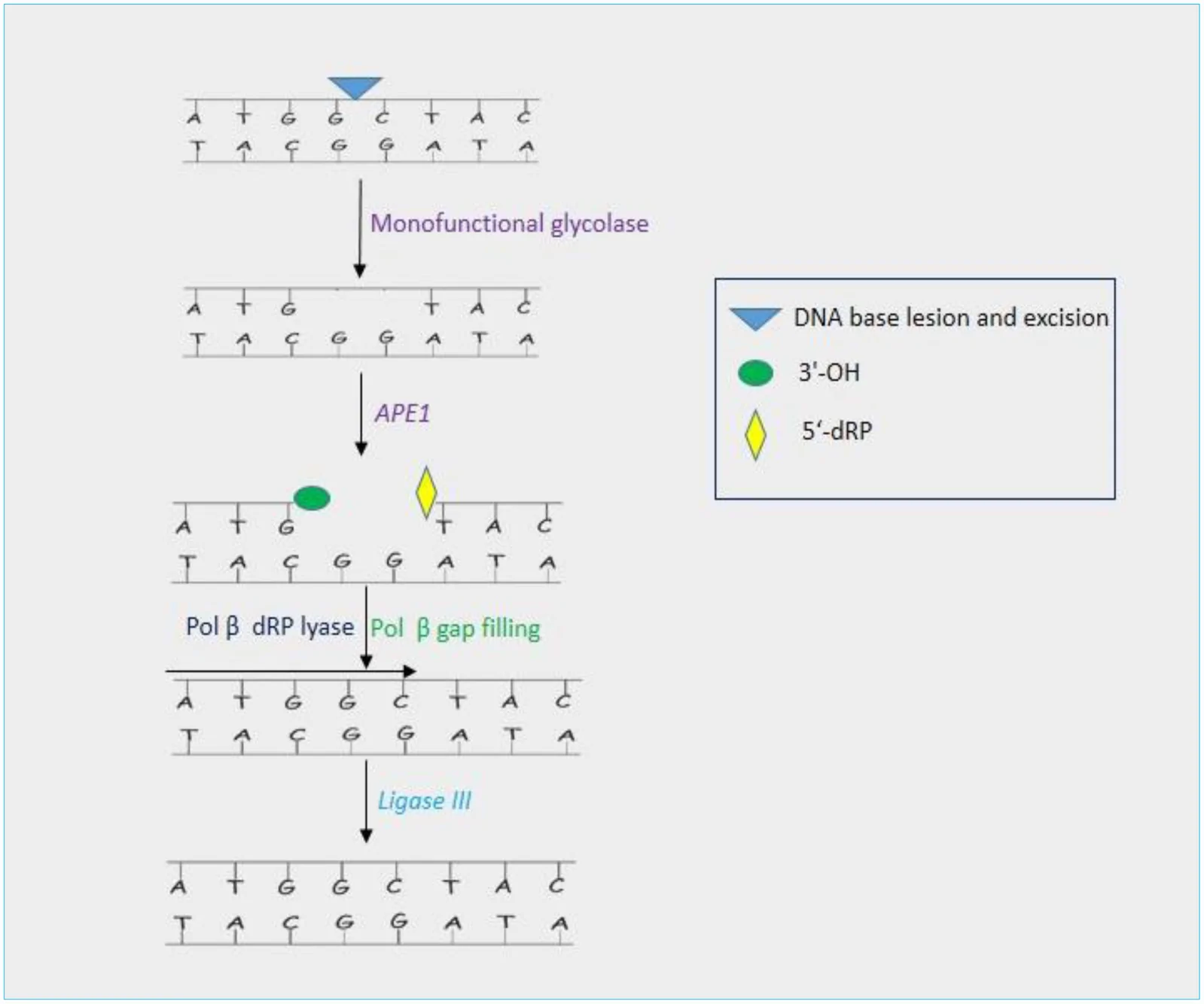
SP-BER DNA repair subpathway. An enzyme called DNA glycosylase excises the broken base, forming an AP site that APE1 processes. Repair pills can reach 3′-OH and 5′-phosphate (5′-P) termini after end-processing. Pol β-mediated single-nucleotide incorporation helps the DNA ligase III complex catalyze strand ligation in SP-BER [40].

OGS and BER are two vital pathways related to oxidative DNA damage that are closely related to the heterogeneity of neurological diseases. For example, MS patients presented increased levels of tert-butyl hydroperoxide (TBH)-induced oxidative stress lesions with a distinct DNA repair pathway, leading to reduced transcript levels of different BER genes, including MBD4 and NTHL1, in MS patients due to single-nucleotide polymorphisms [35],[36]. Moreover, OGS contributes to the accumulation of 5′,8-cyclopurine and 8-oxopurine, resulting in oxidative DNA damage and inducing neurological symptoms [37]. Elevated levels of oxidatively induced DNA damage, particularly 8-hydroxy-2′-deoxyguanosine (8-OH-dG), and abnormalities in the repair of 8-OH-dG by BER have been reported in bipolar disease patients. In this disease, there was also a decreased level of OGG1 and APE1 expression with upregulated POLβ expression, indicating a direct link with OGS-induced oxidative stress and BER damage [38]. In addition to causing DNA damage, OGS has also been reported to be involved in RNA damage in neurological diseases [39]. This pathway is executed by two subpathways: the short-patch (SP-BER) and the long-patch BER (LP-BER) subpathways. However, in this review, we explore and illustrate only the SP-BER pathway. The SP-BER pathway is illustrated in Figure 2, showing the key steps involved in DNA base excision and repair.

8-Hydroxyguanine-oxidized bases, such as 8-oxoG and 7,8-dihydro-8-oxoadenine (8-oxoA), are removed from DNA by DNA glycosylase (OGG1), a bifunctional enzyme [41]–[43]. OGG1 removes 8-oxoG when the base is paired with a natural cytosine (C) but not when it is coupled with native thymine (T). Mammalian cells express at least four OGG1 splice variants, with two playing separate roles in base excision repair (BER) processes that remove 8-oxo-G from the nucleus and mtDNA. An oxidatively damaged base is excised by OGG1, initiating a conventional SP-BER cascade. The mechanism involves enzymes such as APE1, Pol β, and the DNA ligase III complex to restore the original DNA base pair.

#### 2.2.2. Double-strand breaks

Among DNA lesions, DSBs are the most dangerous. To detect, signal, and repair them, DDR signaling—a comprehensive cellular response—is needed. The DDR cannot be carried out without first activating ATM kinase, a protein kinase. The MRE11-RAD50-NBS1 (MRN) complex interacts with it to rapidly recruit to DSB lesions [44],[45]. The phosphorylation of several substrates initiates a signaling cascade and recruits some repair factors to lesions. ATM kinase activity targets serine 139 on the carboxyl terminus of H2AX, also known as γH2AX, in its phosphorylated form. Established γH2AX leads to the activation of ATM and DDR protein accumulation, establishing a positive feedback loop that spreads to a greater extent [46]–[48].

**Figure 3.**
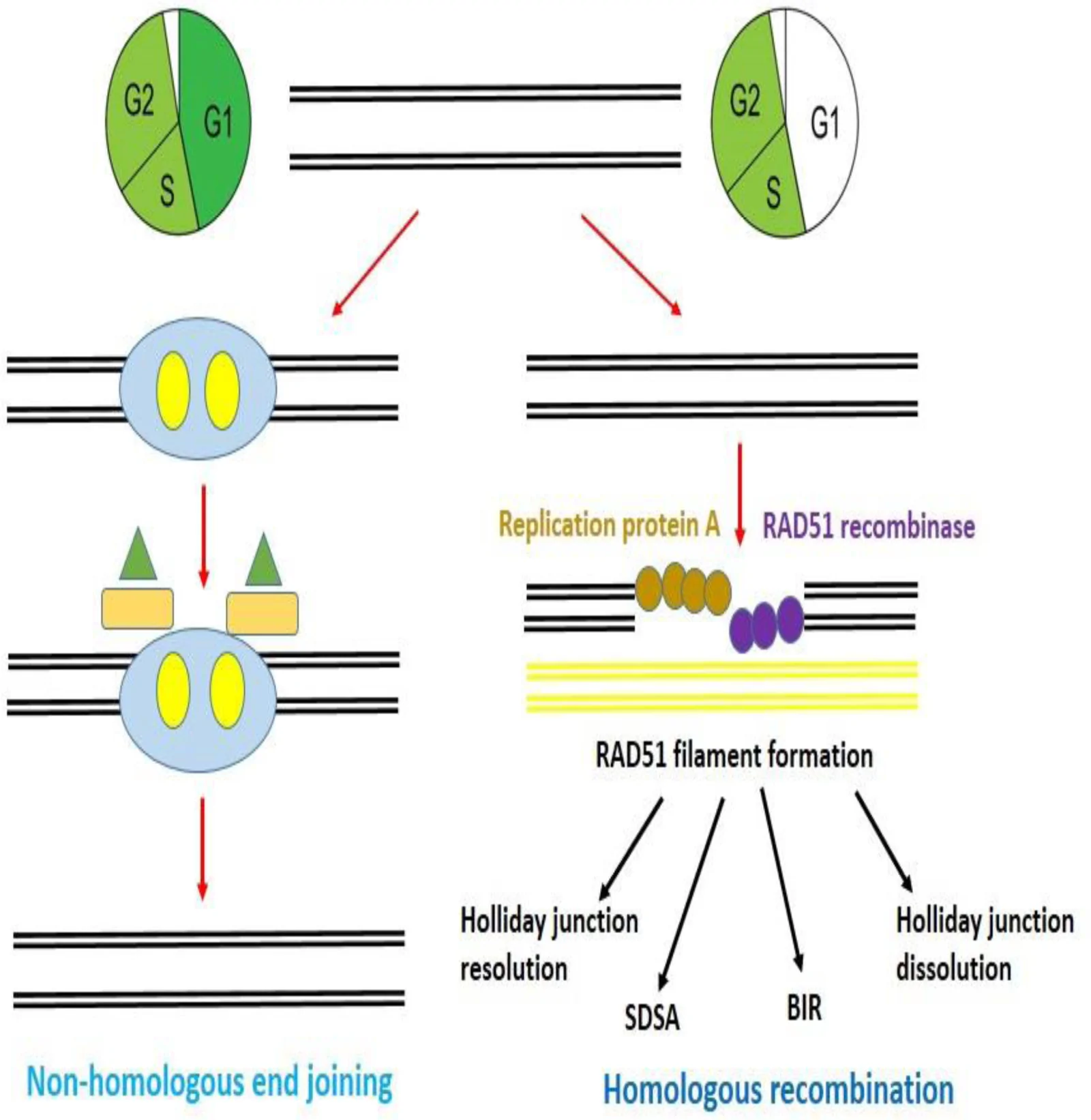
DSB repair pathways. The main phases of NHEJ and HR repair are shown. All four HR pathways—holiday junction resolution, SDSA (Synthesis-Dependent Strand Annealing), BIR (Break-Induced Replication), and dissolution—start with the same stages. The cell cycle strongly influences the selection of DSB repair mechanisms. NHEJ is available during interphase, although HR mechanisms are only available in S/G2 [51].

In terms of DNA strand breaks, there are different ways to encounter repair systems; generally, they can be categorized into two major classes depending on whether a homologous DNA sequence is utilized as a template. When a homologous sequence gap is repaired, nonhomologous end joining (NHEJ) is the method of choice since it involves resealing the two ends of segments directly. Even though it may cause genetic information loss, NHEJ is the most common DSB repair route in most cell lines because it is the simplest and easiest [49]. In contrast to nonhomologous end joining (NHEJ), homologous recombination (HR) requires extensive DNA end processing and uses an identical DNA sequence as a template for repair that is dependent on DNA synthesis [50]. As anticipated, homologous recombination is highly exact, facilitating accurate repair of the damaged locus through the utilization of DNA sequences that are homologous to the broken ends. Homologous recombination primarily utilizes the sister chromatid as a template for the DSB repair pathway, rather than using the identical chromosome as a template. As shown in Figure 3, the main phases of NHEJ and HR repair are illustrated.

## 3. OGS in specific neurodegenerative diseases

### 3.1. OGS leads to amyloid plaques and neurofibrillary tangles in neurons: AD hallmarks

**Figure 4.**
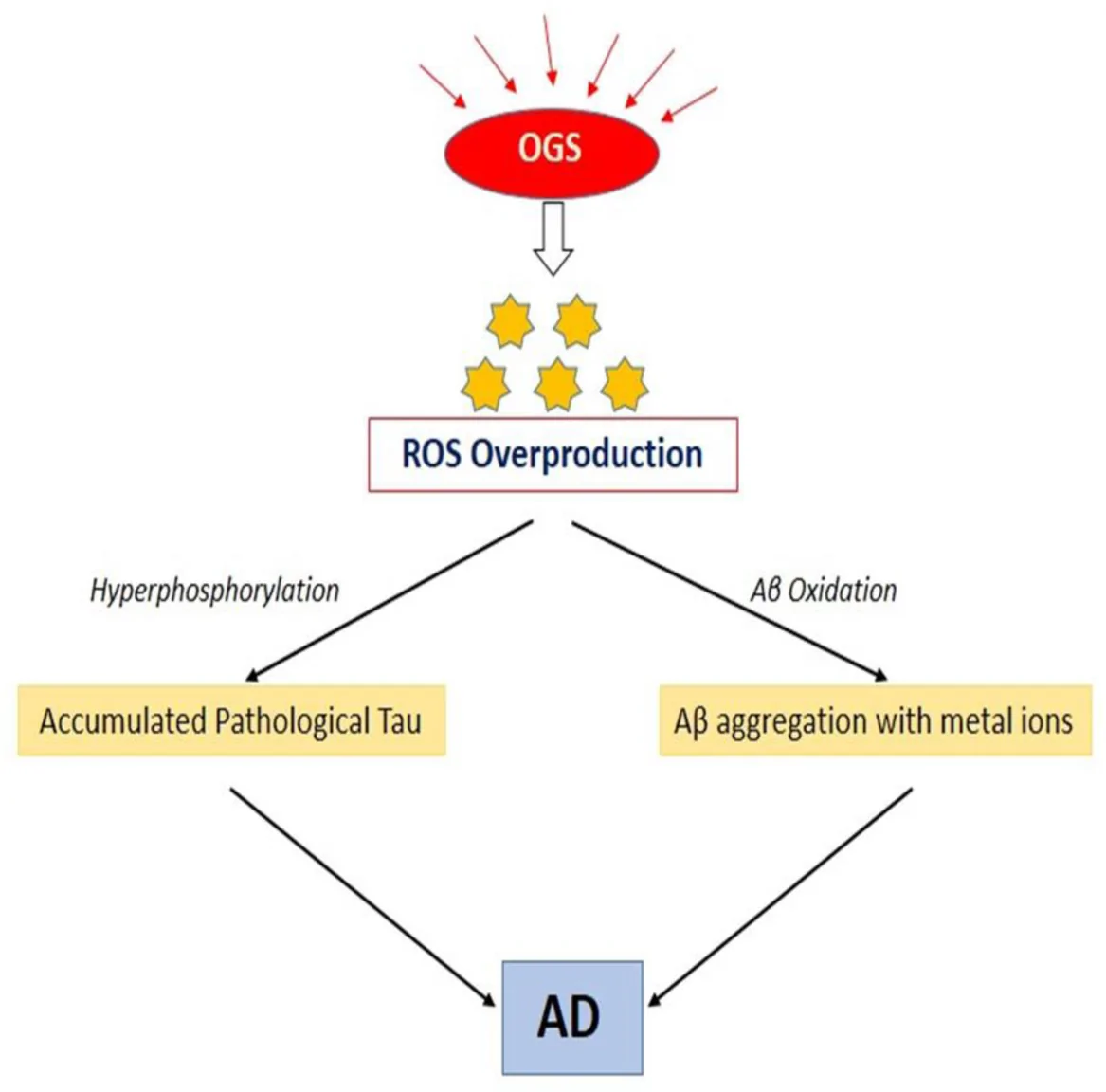
The generation of ROS by genotoxins such as amyloid-β (Aβ) and tau during AD progression.

OGS-induced genomic instability is a key pathological factor in AD, preceding the well-known pathological hallmarks of AD: amyloid plaques and neurofibrillary tangles in AD patients. The pathogenesis of oxidative genotoxicity in AD is a complex and interconnected process involving several intertwined cellular mechanisms, including ROS/OGS-induced cellular damage, mitochondrial dysfunction, and amyloid-beta (Aβ) and tau pathology, leading to a vicious cycle of genomic dysfunctions and neuroinflammation in the AD brain. For example, tau- and Aβ-induced oxidative stress promotes autophagy gene dysregulation, resulting in behavioral disability in AD patients [52]. Moreover, OGS-Aβ networks induce the expression of IKK and NF-κB in neurons, leading to severe neuroinflammation and neuronal death in AD patients [53]. In addition, Aβ and tau pathology have been reported to be involved in autophagy gene dysregulation through oxidative stress during AD pathogenesis. Therefore, these molecular pathways combine to promote AD progression. In contrast, a recent study revealed that the accumulation of tau protein and the resulting dementia are often observed in aged individuals without Aβ deposition, suggesting that tauopathy is a distinct mediator of age-related cognitive decline [54]. In addition, several intracellular molecules, such as circular RNAs (circRNAs), microRNAs (miRNAs), and exosomes (EXOs), regulate extracellular genome function in neurons. A recent review reported different aspects of intracellular molecules and their functions in the pathogenesis of AD [55]. Excessive ROS production triggers mitochondrial dysfunction and neuronal apoptosis, contributing to the pathology of AD, as illustrated in Figure 4.

### 3.2. OGS in the progression of PD: A perfect intracellular storm of cellular damage

OGS is not only an intrinsic part of the pathology of PD but also a side effect. It is intricately linked with mitochondrial dysfunction, dopamine metabolism, α-synuclein aggregation, and neuroinflammation. This complex network initiates a “perfect intracellular storm”, leading to extensive neuronal damage that selectively targets vulnerable dopaminergic neurons, ultimately promoting the debilitating motor symptoms of PD [56],[57]. Understanding this damaging role of OGS is crucial for effective disease-modifying therapies. Recent advances in the pathogenesis of PD and its potential pathways are summarized in Table 1.

**Table 1.** Recent advances have been made in understanding the pathogenesis of PD and its potential pathways.

	**Pathogenesis**	**Pathways**	**Ref.**
Oxidative stress	Dysregulated long non coding RNAs	Aggregation of α-synuclein, mitochondrial dysfunction, calcium stabilization, neuroinflammation	[58]
	Disulfidptosis	Abnormal disulfide bond accumulation, redox imbalance, decreased levels of HSPA9	[59]
	Oxidative damage to lipids, proteins, and DNA	Post mortem PD tissue shows extensive oxidative damage to macromolecules in substantia nigra neurons	[60]
	Mitochondrial dysfunction and ROS-induced apoptosis	Excess ROS mutates mtDNA and triggers caspase activation and mitochondrial-mediated apoptosis via cytochrome C release	[61]
	Impaired antioxidant defenses (e.g., GSH decrease)	Levels of reduced glutathione are significantly lowered in SN, weakening the cell's ability to counteract oxidative stress	[62]
	Iron overload and ferroptosis	Iron accumulation catalyzes ROS generation via Fenton reactions and lipid peroxidation, and promotes ferroptotic cell death	[63]
	Familial PD gene mutations impair oxidative handling	Mutations in *SNCA*, Parkin (*PRKN*), *PINK1*, *DJ-1*, and *LRRK2* disrupt mitochondrial quality control and oxidative stress responses, increasing vulnerability	[64]
	Dopamine metabolism and neuroinflammation contribute to ROS	Dopamine breakdown, neuroinflammation, and microglial activation produce ROS, furthering neuronal damage	[65]

Note: HSPA9: a biomarker of cellular stresses such as glucose deprivation, oxidative injury, ionizing radiation, and caloric restriction [66].

In addition, autosomal recessive early-onset PD is most commonly caused by mutations in the PINK1 (*PARK6*) and Parkin (*PARK2*) genes. They typically manifest clinically with an L-DOPA response, although they may also exhibit dopaminergic-related dyskinesia, hyperreflexia, and, on rare occasions, mental abnormalities. Mutagenic changes in the gene encoding DJ1 (*PARK7*) are another indication of mitochondria-driven parkinsonism. Parkinson's disease (autosomal recessive form), which is less common than PINK1, results from the ensuing loss of function. Individuals with DJ1 genomic changes typically have good L-DOPA responsiveness and slow-progressing PD, which is sometimes accompanied by nonmotor symptoms such as cognitive impairment and psychosis [67].

### 3.3. OGS in HD

The pathogenesis of OGS in HD is a complex process characterized by detrimental cell damage [68]. The precise mechanisms remain under investigation, but recent insights indicate that the mutant huntingtin (mHtt) protein results in an abnormally long polyglutamine (polyQ) tract in the mHtt protein, which is highly prone to misfolding, aggregation, and cleavage into toxic segments [69]. These segments disrupt numerous cellular processes, setting the OGS stage for mitochondrial DNA damage [70]. A well-established marker of this damage is 8-hydroxy-2-deoxyguanosine (8-OHdG), which is present at increased concentrations in the brains of HD mice and patients [71]. In addition, the mHtt protein interferes with the DNA damage response (DDR) and repair mechanisms. Studies have shown that mHtt hampers key DNA repair proteins, such as those involved in the BRCA1 and ATM (ataxia telangiectasia mutated) pathways [69]. Defective DNA repair mechanisms, combined with oxidative damage, can also differentially contribute to the genetic expression of plasticity genes in HD through histone acetylation via impaired localization of CREB-binding protein (CBP) in the HD model [72]. Moreover, OGS-mediated DNA break accumulation abrogates PNKP activity in HD models, resulting in a concomitant decrease in Ataxin-3 activity and promoting CBP ubiquitination and degradation, which adversely impacts transcription and DNA repair [73].

## 4. Physiological and therapeutic strategies for reducing OGS

To slow OGS, mitochondrial work must be targeted [74]. Potential tactics are recommended for the development of interventions that target ROS and prevent mitochondrial fracture to reduce mitochondrial damage and synaptic impairment in AD and PD patients. For example, exercise is highly beneficial for reducing ROS levels and maintaining mitochondrial health. Numerous benefits of working out for people with AD have been reported in other studies [75],[76]. These benefits include the progression of the bloodstream to the brain, increased hippocampal thickness, increased neurogenesis, and improved cognitive work (such as thinking, perceiving, learning, and decision-making).

Another important strategy is to consume sufficient amounts of vitamins and minerals to maintain a solid antioxidant status and to utilize organic foods high in cancer-prevention agents. Vitamin C rich nourishment can help diminish ROS [77]. The most frequently utilized antioxidant in clinical and research facilities is vitamin C, which can be given at diverse concentrations, intensely or chronically, alone or in combination with other cancer-prevention agents [78]. Innovative pharmaceutical approaches are considered the best options for alleviating OGS. Among these substances, biochemical factors, such as coenzyme Q10, idebenone, amino acid compounds, mitochondrial supplements, Mito VitE, sulforaphane, synthetic dyes, curcumin, and organic fatty acids, act on PGC-1α and activate mitochondrial biogenesis [79]. These substances offer advantages such as safeguarding mitochondrial function, decreasing ROS, and enhancing bioenergetics. The therapeutic viability of these mitochondrion-targeted drugs is substantiated by certain preclinical evidence. For example, the genetic overexpression of PGC-1α in transgenic animal models of AD has been shown to increase mitochondrial dynamics while decreasing the production of Aβ via BACE1 suppression. Therefore, pharmaceutical agents such as bezafibrate, metformin, and others that promote mitochondrial biogenesis are capable of achieving this effect.

Moreover, CoQ10, a crucial element of the electron transport chain (ETC), mitigates oxidative stress and neurodegenerative diseases in neuronal cells by preserving mitochondrial ∆Tm, enhancing ATP synthesis, and decreasing ROS production [80]. Furthermore, it enhances mitochondrial mass and bioenergetic function while safeguarding the phospholipid bilayer and mitochondrial proteins from oxidative damage [81]. Another finding indicates that the regular intake of CoQ10 significantly enhances the activity of antioxidant proteins and reduces inflammation [82]. Several therapeutic approaches have been identified for treating neurodegenerative diseases, as summarized in Table 2.

**Table 2.** Therapeutic approaches for treating OGS.

**Therapeutic approach**	**Agent/tool**	**Mechanism**	**Status/trial**	**Efficacy**	**Safety**	**Ref.**
Antioxidants	Vitamin C, CoQ10	ROS scavenging, mt protection	Preclinical	Shown to reduce ROS and improve mitochondrial functions in models	Generally, safe at physiological doses; high-dose CoQ10 may cause GI symptoms	[79]
DNA repair boosters	NAD + precursors	Enhances PARP activity, supports repair of oxidative DNA lesions	Preclinical	Promising in enhancing DNA repair and mitochondrial function in vitro	Good safety profile; mild flushing and nausea reported at high doses	[83]
Gene therapy	CRISPR/Cas9	Gene editing in AD/PD models	In vivo mice	High precision targeting; promising neuroprotective effects in animal models	Risks of off-target effects and immune responses in vivo	[84]–[86]
Hormesis-based therapies	Mild oxidative stressors	Induces adaptive antioxidant defenses	Conceptual/early stage	Theoretical benefits; animal models show enhanced stress resilience	Dose-dependent risks; excessive stressors can be harmful	[87]
Lifestyle	Exercise, diet	Enhances mitochondrial health	Clinical evidence	Strong evidence for reducing oxidative burden and improving cognition	Safe; may vary based on patient condition and adherence	[88]
Mitochondrial biogenesis	PGC-1α activators	Enhances ATP, reduces ROS	Early-stage	Effective in improving mitochondrial biogenesis in cell and animal studies	Needs more human data; some agents show metabolic effects	[79]

## 5. Emerging technologies, advantages and future directions

### 5.1. CRISPR/Cas9-based gene-editing technology

One of the most cutting-edge molecular technologies is CRISPR/Cas9-based gene-editing technology [84]. CRISPR offers a powerful approach for treating AD and PD diseases by directly addressing their genetic roots. It can precisely correct disease-causing mutations, such as those in genes related to beta-amyloid plaques in AD patients or alpha-synuclein in PD patients. This method has the potential to not only manage symptoms but also halt or reverse the progression of these devastating neurodegenerative disorders. Moreover, the ability of CRISPR/Cas9 to efficiently cut double-stranded DNA was the primary emphasis of early studies. Flat ends can be produced via DSBs when the sgRNA directs Cas9 to a particular site and when there are nuclease structural domains for HNH and RuvC. This process activates DNA repair mechanisms, the two most important of which are NHEJ and HDR [85],[89]. Therefore, it is possible that diseases could be treated in the laboratory as well as in the clinic through the delivery of CRISPR/Cas systems to targeted areas of the body. Because of its great precision, efficacy, and ease of handling, it is expected to be one of the most sought-after technologies in the years to come. Nevertheless, while CRISPR technology is employed to modify genes, researchers have reported that some circumstances are not anticipated [84].

### 5.2. Biological indicators of oxidative DNA loss

Another highly effective technology uses biological indicators of oxidative DNA loss in cerebrospinal fluid (CSF) or blood, which are primarily 8-OHdG and 8-oxo-7,8-dihydro-2′-deoxyguanosine (8-oxodG). Thus, these markers are widely used to measure oxidative stress. Research shows that urine 8-OHdG can predict cancer and degenerative disease risk. The main quantitative measurement methods are HPLC with electrochemical detection (EC) and HPLC tandem mass spectrometry, among others [90],[91].

### 5.3. Various cell-based regeneration and rejuvenation strategies

Age-related disorders that significantly impair quality of life and place a heavy burden on society include neurodegenerative diseases, such as AD and PD. A major contributing element to the onset and progression of these diseases is cellular senescence, which affects different types of brain cells and promotes permanent cell cycle arrest and reduced cellular activity. R3 strategies—rejuvenation, regeneration, and replacement—have been emphasized as viable therapeutic options for treating neurodegeneration in recent advances in regenerative medicine. Stem cell therapy, direct lineage reprogramming, and partial reprogramming in the context of R3 emphasize how these interventions mitigate cellular senescence and counteract aging-related neurodegeneration [92].

### 5.4. Multitarget drug design for the treatment of AD

Traditional single-target medications have had poor therapeutic success; they have not been able to stop, cure, or reverse the course of neurodegenerative diseases such as AD. As a result, multitarget drug design (MTDD) and other comprehensive therapeutic approaches are necessary for this complex disease. Targeting several disease pathways simultaneously with MTDD is a potential tactic. The accuracy and efficacy of MTDD can be further improved by integrating cutting-edge technologies such as artificial intelligence, machine learning, and nanomedicine. The key benefits of MTDD include increased treatment scope, pathway-level synergy, and the possibility of increased efficacy [93].

### 5.5. Stem cell–derived extracellular vesicles in neurodegenerative diseases

Stem cell–derived extracellular vesicles (EVs) act as nanocarriers that reprogram diseased neural circuits chiefly by resolving neuroinflammation, restoring proteostasis, and supporting neuronal repair [94]. In AD and PD models, MSC-/NSC-EV miRNAs and proteins suppress NF-κB/NLRP3 signaling, enhance autophagy-lysosomal pathways (including neprilysin-mediated Aβ degradation), and improve mitochondrial resilience and synaptic plasticity. Collectively, EVs offer a cell-free, engineerable alternative to stem-cell transplantation, although standardization, GMP (Good Manufacturing Practice) scale-up, and long-term safety still need to be solved before routine clinical use [95].

### 5.6. Nanoparticles coated with exosomes to treat neurodegenerative diseases as biomarkers and therapeutic agents

Complex neurobiological modifications, which manifest as biomarker changes in blood, cerebrospinal fluid (CSF), and brain imaging, are hallmarks of neurodegenerative diseases [96]. For example, exosome or exosome-coated nanoparticles (NPs) combine the multifunctionality of NPs with the inherent qualities of exosomes. Exosomal membranes facilitate blood–brain barrier penetration and provide microRNA- and protein-mediated neuroprotection, whereas the nanoparticle core enables the sustained release of therapeutic payloads such as antioxidants, siRNAs, or dopamine [97]. Therefore, the use of exosomes coated with NPs may improve the accuracy, effectiveness, and safety of therapeutic interventions for treating neurodegenerative disease [98],[99].

### 5.7. Neurotrophic genes that target neurodegenerative disorders

Neurodegenerative illnesses (NDDs) such as AD, PD, and HD have demonstrated considerable potential for gene therapy as a viable therapeutic intervention. Gene delivery of NTF has the potential to be used as a therapeutic approach for the treatment of neurological problems in the brain [100]. Neurotrophic genes, including *BDNF*, *GDNF*, and *NGF* and their receptors, play central roles in controlling neurodegenerative diseases by activating survival pathways (PI3K/Akt, MAPK/ERK), enhancing synaptic plasticity, stimulating neurogenesis, and suppressing neuroinflammation. Their products also promote the clearance of toxic aggregates such as amyloid-β, tau, and α-synuclein, thereby preserving neuronal integrity [101].

## 6. Conclusions

Oxidative genotoxic stress (OGS) serves as a crucial pathogenic element in the onset and progression of neurodegenerative diseases, such as AD, PD, and HD. Neurons, characterized by their significant metabolic requirements, extended lifespan, and restricted ability to regenerate, are extremely vulnerable to oxidative genomic damage caused by ROS and RNS. We have shown that OGS, arising from an imbalance of reactive oxygen species and antioxidant defenses, initiates a cascade of detrimental events. These include significant DNA damage, such as genomic instability and epigenetic alterations, as well as the impairment of vital cellular processes such as mitochondrial function and DNA repair. The resulting neuroinflammation and neuronal dysfunction establish a vicious cycle that drives disease progression. Given the complex and multifaceted nature of OGS-mediated neurodegeneration, it is not surprising that traditional single-target therapeutic approaches have yielded limited success. This work highlights a paradigm shift toward a comprehensive, multitargeted strategy. We explored a range of promising avenues, from lifestyle modifications and potent antioxidant therapies to advanced pharmaceutical interventions such as mitochondrial-targeted drugs. Furthermore, we discuss the potential of emerging technologies, including CRISPR/Cas9 gene editing and innovative drug delivery systems such as exosome-coated nanoparticles, to directly counteract the molecular underpinnings of this disease. Ultimately, a deeper and more integrated understanding of these intricate molecular pathways is essential for the future development of effective treatments. This study provides insights into the mechanisms of OGS and the assessment of novel therapeutic strategies, paving the way for a more holistic approach to combating neurodegenerative diseases. Despite the insights discussed, this review has certain limitations. First, much of the evidence linking oxidative genotoxic stress to neurodegeneration is derived from preclinical or in vitro studies, which may not fully capture the complex human disease environment. Second, inconsistencies in experimental models, biomarker specificity, and methodological variability across studies limit the ability to generalize findings. Third, the lack of large-scale longitudinal clinical data makes establishing causality between oxidative genomic damage and disease progression difficult. Finally, while therapeutic strategies such as antioxidants, mitochondrial enhancers, and genome-editing technologies show promise, their translation into effective clinical interventions remains largely unproven and requires rigorous validation through well-designed clinical trials.

## Use of AI tools declaration

The authors declare they have not used Artificial Intelligence (AI) tools in the creation of this article.
